# Comparison of Airway Responses Induced in a Mouse Model by the Gas and Particulate Fractions of Gasoline Direct Injection Engine Exhaust

**DOI:** 10.3390/ijerph15030429

**Published:** 2018-03-01

**Authors:** Caitlin L. Maikawa, Naomi Zimmerman, Manuel Ramos, Mittal Shah, James S. Wallace, Krystal J. Godri Pollitt

**Affiliations:** 1 Environmental Health Sciences, University of Massachusetts, 686 North Pleasant Street, Goessmann Laboratory Room 175, Amherst, MA 01003, USA; cmaikawa@stanford.edu; 2 Mechanical Engineering, University of British Columbia, Vancouver, BC V6T 1Z4, Canada; nzimmerman@mech.ubc.ca; 3 Mechanical and Industrial Engineering, University of Toronto, Toronto, ON M5S 3G8, Canada; manuel.ramos@mail.utoronto.ca (M.R.); wallace@mie.utoronto.ca (J.S.W.); 4 Institute of Orthopaedics and Musculoskeletal Sciences, University College London, Royal National Orthopaedic Hospital, Stanmore HA7 4LP, UK; mittal.shah@ucl.ac.uk

**Keywords:** gasoline direct injection engine exhaust, polycyclic aromatic hydrocarbons, inflammation, *Cyp1b1*, *TNFα*, in vivo

## Abstract

Diesel exhaust has been associated with asthma, but its response to other engine emissions is not clear. The increasing prevalence of vehicles with gasoline direct injection (GDI) engines motivated this study, and the objective was to evaluate pulmonary responses induced by acute exposure to GDI engine exhaust in an allergic asthma murine model. Mice were sensitized with an allergen to induce airway hyperresponsiveness or treated with saline (non-allergic group). Animals were challenged for 2-h to exhaust from a laboratory GDI engine operated at conditions equivalent to a highway cruise. Exhaust was filtered to assess responses induced by the particulate and gas fractions. Short-term exposure to particulate matter from GDI engine exhaust induced upregulation of genes related to polycyclic aromatic hydrocarbon (PAH) metabolism (*Cyp1b1*) and inflammation (*TNFα*) in the lungs of non-allergic mice. High molecular weight PAHs dominated the particulate fraction of the exhaust, and this response was therefore likely attributable to the presence of these PAHs. The particle fraction of GDI engine exhaust further contributed to enhanced methacholine responsiveness in the central and peripheral tissues in animals with airway hyperresponsiveness. As GDI engines gain prevalence in the vehicle fleet, understanding the health impacts of their emissions becomes increasingly important.

## 1. Introduction

Asthma is a chronic disease with increasing prevalence in North America [1]. Genetic and environmental factors have been attributed to the development and exacerbation of asthma. Allergen exposure is a prevalent environmental factor associated with the onset of allergic asthma, which is characterized by airway inflammation and mucus production, resulting in obstruction and hyperresponsiveness of the airways.

Air pollutants have also been explored as an environmental factor that contributes to increased asthma risk [2]. Diesel exhaust particulate matter (PM) has been extensively used by studies investigating the mechanism by which air pollutant exposure affects asthma. While this particle type represents a large component of traffic-related air pollution, the vehicle fleet is changing to adapt to increasingly strict emissions standards. PM emissions are being regulated to 3 mg/mile in the United States as of 2017 [3], and California Low Emission Vehicle (LEV) III standards are proposing to further reduce regulations to 1 mg/mile in 2025 [4]. European regulations are currently 5 mg/km [5]. Gasoline direct injection (GDI) engines are becoming more prevalent, with US GDI engine market shares increasing from 4% to 42% from 2009 to 2015 [6]. The market shares of GDI engines are expected to reach 93% by 2025 [7]. The trend in the US market reflects global GDI engine implementation in order to address the increases in fuel economy standards. The effect of GDI engine exhaust exposures on asthma has not yet been evaluated.

Compared to traditional gasoline port injection fuel engines, GDI engines have improved fuel efficiency and similar nitrogen oxide, carbon dioxide, and carbonyl emission levels [8,9]. However, GDI engines are characterized by higher particulate matter concentrations [9,10,11,12], including black carbon [9,10]. Recent studies have shown that GDI engines can generate PM emissions that exceed current emissions regulations [9,13].

Many studies have examined whole diesel exhaust or isolated the particulate fraction of the exhaust. However, semi-volatile organics, such as polycyclic aromatic hydrocarbons (PAHs) released in engine exhaust, are distributed between the gas and particulate phases. Phase partitioning is dependent on the vapour pressure of the compound and ambient temperature, as well as on the particle surface area. At elevated vehicle exhaust temperatures, PAHs exist predominately in the gas phase [14]. Consideration of a combination of the gas and particulate fractions in the raw GDI engine exhaust is critical when evaluating the capacity of traffic-related air pollutants to exacerbate asthma.

Once inhaled, PAHs are metabolically activated by cytochrome P450 (CYP) enzymes, primarily CYP1B1, to oxygenated metabolites with enhanced reactivity [15]. These oxygenated metabolites have the capability to produce reactive oxygen species (ROS), which can lead to an inflammatory airway response [16]. PM derived from diesel engine exhaust has been previously used to evaluate airway mechanics or an inflammatory response in both allergic and non-allergic mouse models. Increased airway resistance after diesel engine particulate matter or exhaust exposure was observed in non-allergic mouse models [17,18].

This study investigated exposure concentrations of gaseous and particulate PAHs emitted in GDI engine exhaust. Our objective was to contrast the capacity of the particulate compounds with the gas fraction of vehicular exhaust to induce pulmonary responses in naïve mice, as well as in animals with airway hyperresponsiveness. We hypothesized that PAH activation of cytochrome P450 enzymes was a molecular link between GDI exhaust exposure and the induction of airway inflammation.

## 2. Materials and Methods

### 2.1. Mice

Female Balb/c mice (aged 8–10 weeks; mean body weight, 19.4 g) obtained from Charles River Laboratory were used in this study. Females were chosen for this study, as they are more widely used due to the convenience in husbandry of female mice, which is better tolerated than in males. Additionally, at this stage we do not predict a bias towards gender in our redox responses. It may be important in future studies to investigate the gender differences in immune functional readouts; however, it is not within the scope of this this study at this stage.

Animal protocols were approved by the University of Toronto Faculty Advisory Committee on Animal Services and were conducted in accordance with the guidelines of the Canadian Council on Animal Care.

### 2.2. Murine Model of Allergic Airway Inflammation

Mice were sensitized to house dust mite (HDM; *Dermatophagoides pteronyssinus*, Greer Laboratories Inc. Lenoir, NC, USA) to elicit allergen-induced airway inflammation (Figure S1). We followed an established acute exposure model, which included five consecutive daily intranasal exposures (25 μg of crude extract in 35 μL normal saline) followed by a single intranasal sensitization 7 days later [19,20,21]. Disease induced by this acute exposure model has been previously demonstrated to manifest primary phenotypes of airway hyperresponsiveness [22]. Control groups were instilled intranasally with 35 µL of saline solution. HDM and saline sensitized mice were exposed to GDI engine exhaust on the day following the final intranasal sensitisation.

### 2.3. Engine Exhaust Characterisation

**Figure 1:**
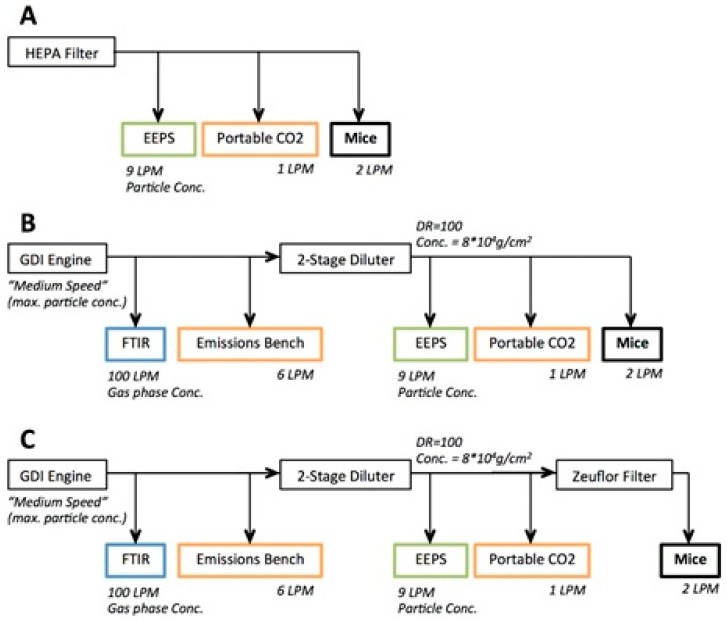
Overview of GDI engine exhaust sampling and exposure system: HEPA filtered ambient air (**A**); GDI engine exhaust (**B**); and GDI engine exhaust filtered with the Zefluor filter filtered GDI (**C**).

**Figure 2:**
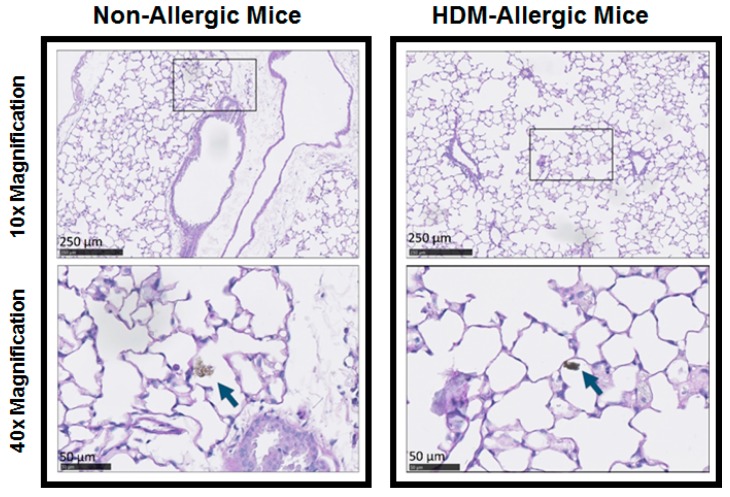
Deposition of agglomerate particles in alveolar ducts following exposure to GDI engine exhaust. Deposition was observed in saline- (left) and HDM (right)-treated animals. Particle agglomerates are indicated by blue arrows. High power images (40× magnification) are representative of the boxed regions in the low power images (10× magnification).

Figure 1 shows an overview of the exposure system. GDI engine exhaust for exposures was generated by a light-duty engine (2012 Ford Focus, 2.0 L displacement). A rotating disc thermodiluter (TSI 379020A) was used to dilute the exhaust at a dilution ratio of 100. All concentrations are reported as diluted concentrations. The diluted exhaust (dilution ratio: 100; 8 × 10^4^ particles/cm^3^) was fed into the exposure chamber and the engine exhaust particle sizer (EEPS, TSI 3090, The chauvet hotel, MN, USA); the EEPS was used to confirm that particle number concentrations were consistent across exposures. Carbon dioxide measurements were taken upstream (MKS 2030HS FTIR, Andover, MA, USA) and downstream (LICOR 840A, Lincoln, NE, USA) of the diluter to ensure consistent dilution ratios across the measurement campaign. The engine was run at steady-state conditions that were representative of highway cruise conditions (2600 rpm, 56 Nm) at a speed of 100 km/h. Commercially-available premium gasoline was used to fuel the engine. The gasoline contained no ethanol. There was no exhaust aftertreatment on the engine, and measurements were sampled from engine-out exhaust. The exhaust temperature and humidity were decreased by the thermodilution and were at ambient levels upstream of the animal exposure chamber. A 47 mm Zefluor filter (Pall Corporation, Port Washington, WA, USA) was placed upstream of the exposure chamber for some exposures to isolate the pulmonary response to the gaseous fraction of the exhaust. Three exposure scenarios were tested: (1) control exposure with HEPA filtered ambient air, Figure 1A; (2) GDI engine exhaust, Figure 1B; and (3) GDI engine exhaust filtered with the Zefluor filter filtered GDI (f-GDI), Figure 1C. Particle size distribution of the GDI engine exhaust was characterized using the EEPS. PAH concentrations were also evaluated using a filter pack fed with GDI engine exhaust diluted by a Dekati Fine Particle Sampler (FPS-4000, Dekati Inc. Kangasala, Finland, dilution ratio = 15) and passed through a PM_2.5_ cyclone. Zimmerman et al. [23] have described this set up and its operation in detail. Briefly, a pre-fired 47 mm quartz filter (Pali Corporation, Port Washington, WA, USA) and a XAD-4 coated quartz filter (sorbent-impregnated filter, SIFs, Rohm & Haas, Philadelphia, PA, USA) were contained in a filter pack. Samples through the filter pack were taken over a 20-min period with a flow of 26 L/min. After collection, SIFs and quartz filters were stored in an airtight container in a freezer (<−4 °C), in 47 mm petri dishes (Pall Corporation, Port Washington, WA, USA).

An isotopically-labelled internal standard mixture was used to spike filters before extraction. High pressure liquid chromatography grade dichloromethane was used for filter extraction within two weeks of sample collection. Mild sonication at room temperature was used for extraction. Pre-cleaned glass wool and sodium sulphate were used to filter sample extracts and nitrogen gas was used to concentrate the samples [24]. A gas chromatography mass spectrometry system (Agilent GC-6890N plus MSD-5973N) fitted with a HP-5MS column (30 m, 0.25 mm diameter, 0.25 μm thickness) was used for analysis of the sample extracts. A five-point standard curve of pure natural compound standards, ranging from 20 to 1000 pg/μL for each PAH congener, was used to assess the concentrations of extracts.

### 2.4. GDI Engine Exhaust Inhalation Exposure

A modified inExpose nose-only inhalation system (Scireq Inc., Montreal, PQ, Canada) was used to expose the mice to GDI engine exhaust emissions for 120 min. Exposures were conducted using an exposure chamber built specifically for mouse pollutant exposures; it has been used previously by this group for ambient air pollution exposure studies [22]. Each non-allergic and HDM-allergic group included *n* = 8 mice exposed to HEPA filtered air exposures, *n* = 8 mice for the GDI engine exhaust exposures, and *n* = 9 mice for the f-GDI engine exhaust exposures.

### 2.5. Measurement of Airway Hyperresponsiveness and RNA Isolation from Murine Lungs

Immediately after the exposure period, the flexiVent (Scireq Inc., Montreal, PQ, Canada) was used for pulmonary function testing and assessment of methacholine responsiveness. Ketamine and xylazine (10 mg/kg and 50 mg/kg body weight, respectively) were used to anesthetise the mice prior to testing. Mice were also treated via intraperitoneal administration with a muscle paralytic (pancuronium; 0.3–0.8mg/kg) during pulmonary function testing, to prevent any respiratory drive artefact, which would prevent obtainment of reliable pulmonary function data. The trachea was cannulated with an 18-gauge cannula. Mice were then tested for responsiveness to a methacholine challenge (0.01–100 mg/mL) by direct nebulisation of methacholine into the ventilator circuit. The maximal response of total respiratory resistance, Newtonian central airways resistance, and peripheral tissue damping (related to tissue resistance) was taken at each dose [22]. Pulmonary function tests alternated between HDM and saline sensitized mice to equalize variation in time between exposure time and tests. Immediately, after the pulmonary function data was collected, blood sampling by cardiac puncture was conducted. Mice were then euthanized and lung tissue was harvested from the mice for histological (*n* = 3 for HEPA filtered air and f-GDI explores; *n* = 4 for the GDI exposure) and gene expression (*n* = 5) analyses.

### 2.6. Histology Analysis

Lung tissues were excised and fixed with 4% paraformaldehyde solution (Canemco, Gore, QC, Canada), and wax sections were cut. Serial 4µm longitudinal sections were cut parallel to the main bronchus across the left lobe. The longitudinal sections were stained with a Hematoxylin and Eosin, Masson Trichrome, and Periodic acid–Schiff–diastase at the Centre for Modelling Human Disease (Toronto Centre for Phenogenomics, Toronto, ON, Canada). Images were then visualized on an inverted microscope (Leica DMIL) with an attached camera (Olympus DP71, Waltham, MA, USA). The mice exposed to HEPA filtered air were used as a control for both saline and HDM groups.

### 2.7. Assessment of Quantitative PCR in Airways

Total RNA was isolated using the PureLink RNA Mini Kit (Life Technologies, Carlsbad, CA, USA). Homogenisation of the whole lung sections to isolate RNA was performed with a rotor-stator homogenizer. UV-vis spectroscopy absorbance was used to evaluate RNA integrity [25]. cDNA was transcribed from one microgram of the total RNA according to the manufacturer’s instructions using the SuperScript VILO cDNA synthesis kit (Invitrogen, Carlsbad, CA, USA). A cycle of 10 min mixing and incubation at 25 °C, 60 min incubation at 42 °C, and 5 min incubation at 85 °C to terminate the reaction was performed in the ThermoCycler (BioRad C100, Hercules, CA, USA) to synthesize cDNA. LightCycler^®^ 480 Probe Master and Primer-probe mix were combined with 2.5 μL cDNA for a total reaction volume of 10 μL. Real-time quantitative PCR using a Sequence Detection System (Applied Biosystems Prism 7900HT, Foster City, CA, USA) was performed using TaqMan Gene Expression Assays (Life Technologies, Carlsbad, CA, USA) to determine mRNA levels for the genes of interest. Genes of interest included cytochrome p450 1B1 (*Cyp1b1*; mCG12056), CXC chemokine (*Cxcl1*; mCG1708), and tumor necrosis factor alpha (*Tnfα*; mCG15911). The 2^−ΔΔCt^ method described by Schmittgen and Livak [26] was used to determine target gene quantification relative to the housekeeping gene (*Ppia,* Gm17494). *Ppia* was chosen on the basis that PPIase has been shown to be more consistently expressed in various mouse tissues compared to the more commonly used GAPDH or Actin. Furthermore, prior studies have implicated PPIase as a suitable housekeeping control [27,28]. The non-allergic mice exposed to HEPA filtered air were used as the control for reporting relative fold change.

### 2.8. Statistical Analyses

All results are presented as the mean and the associated standard deviation (SD). All measured outcome variables (pulmonary function, histology, and gene expression) were contrast between two animal groups (non-allergic mice and HDM-allergic mice) and three exposure groups (HEPA filtered air, GDI engine exhaust, and filtered GDI engine exhaust). Statistical analyses of pulmonary function data have been previously described [22,29,30]. Differences in individual doses of methacholine measured for dose-response curves of total respiratory system resistance, Newtonian central airways resistance, and peripheral tissue damping were compared using a two-way ANOVA. To compare maximum methacholine responsiveness of total respiratory system resistance, Newtonian central airways resistance, and peripheral tissue damping, one-way ANOVA with Bonferroni comparisons was tested to compare the exposure groups. Gene expression results were similarly analysed across exposure groups using a one-way ANOVA with Bonferroni post-hoc testing. *p*-values < 0.05 were considered significant for all analyses. All statistical tests were performed using SAS 9.3 (Cary, Wake county, NC, USA).

## 3. Results

### 3.1. Characterisation of GDI Engine Exhaust Exposure Concentrations

Mouse exposure concentrations were 100 times lower than exhaust concentrations following dilution. This dilution factor was selected to align exposures with levels of traffic-related air pollutant that are representative of concentrations measured in Toronto, Canada [31]. The characteristics of the GDI and f-GDI engine exhaust used for animal exposure in this study have been previously described [32].

Briefly, the GDI engine exhaust exposures were characterized by a mean total particle concentration of 4.6 × 10^4^ particles/cm^3^ (SD = 1.3 × 10^3^). The mean particle size was 75 nm for the GDI engine exhaust. Filtration of the GDI engine exhaust with a Zefluor efficiently removed particles such that the mean total particle concentration for this exposure scenario was 4.2 × 10^3^ particles/cm^3^ (SD = 5.1 × 10^2^). The total particle concentration was further decreased for the HEPA filtered air exposure group (<180 particles/cm^3^, SD = 60).

Individual concentrations of PAHs characterized have been previously reported [32]. High molecular weight PAH species were primarily in the particulate phase, and low molecular weight species were found in the gas phase. Approximately 85% of total measured PAH concentration was composed of low molecular weight species. These low molecular weight species in engine exhaust exposures were dominated by acenaphthylene (232 ng/m^3^, SD = 79), phenanthrene (565 ng/m^3^, SD = 120), and anthracene (310 ng/m^3^, SD = 51).

The GDI engine exhaust and f-GDI engine exhaust exposure groups were characterized by the equivalent gaseous concentrations [32]. The GDI engine was operated without a normal 3-way catalytic converter and yielded elevated carbon monoxide exhaust concentration (65 ppmv, SD = 4.0). The mean concentration of nitrogen oxide across all engine exhaust exposures was 20 ppmv (SD = 1.0), and the mean formaldehyde concentration was 0.87 ppmv (SD = 0.05).

### 3.2. Murine Model of Allergic Airways Inflammation

Pulmonary function testing demonstrated the expected increase in the methacholine responsiveness in HDM-allergic mice compared to non-allergic controls for measurements of total respiratory system resistance (*p* < 0.001), Newtonian central airways resistance (*p* < 0.001), and peripheral tissue damping (*p* < 0.001) (Figures S2–S4).

### 3.3. Single-Compartment Model of Respiratory Mechanics

Total maximum resistance was increased for HDM-allergic animals relative to non-allergic animals across all exposure types in response to methacholine (100 mg/mL) (Table 1). Control non-allergic mice did not exhibit increased total resistance or total maximum resistance following GDI engine exhaust or f-GDI engine exhaust exposure, relative to HEPA filtered air. In contrast, HDM-allergic animals exhibited elevated total airway resistance when exposed to GDI engine exhaust (*p* = 0.023) and f-GDI engine exhaust (*p* = 0.021) relative to HEPA filtered air at the maximum methacholine dose. No difference in total airway resistance was observed between GDI and f-GDI engine exhaust exposures.

### 3.4. Constant-Phase Model of Respiratory Mechanics

Airway hyperresponsiveness was delineated into enhanced respiratory resistance in the central airways (Newtonian resistance) and the peripheral tissues (dampening). Non-allergic mice did not exhibit any change in methacholine response in the central airways following GDI or f-GDI engine exhaust exposures compared to HEPA filtered air (Table 1). In contrast, an enhanced maximal central airway resistance was observed in the HDM-allergic group to GDI engine exhaust compared to the HEPA filtered air (*p* = 0.037) and f-GDI exposure (*p* = 0.042). f-GDI engine exhaust exposures did not induce any change in the maximum resistance response of the central airways compared to the HEPA filtered air exposure.

In the peripheral tissue, increased dampening was found for HDM-allergic mice after exposure to GDI engine exhaust compared to HEPA filtered air (*p* = 0.036; Table 1). A trend towards increased maximum dampening in the peripheral tissues after f-GDI engine exhaust exposed HDM-allergic mice relative to HEPA filtered air was also observed (*p* = 0.07). There was no difference in peripheral tissue air resistance observed in non-allergic mice across exposure groups.

### 3.5. Histology of Lung Tissue

**Figure 3:**
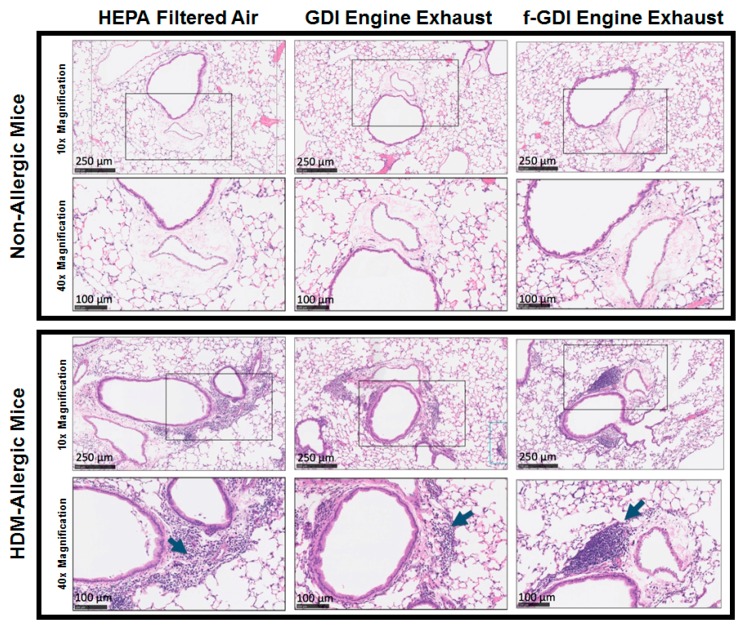
Enhanced inflammation induced by sensitisation to HDM. Hematoxylin and Eosin staining demonstrated increased peribronchial and perivascular inflammatory infiltrates in HDM-allergic animals as compared to non-allergic mice. Cell infiltration is indicated by blue arrows. High power images (40× magnification, Rows 2 and 4) are representative of the boxed regions in the low power images (10× magnification, Rows 1 and 3). These images are representative samples of the three exposure groups.

Deposition of GDI particles was observed in alveolar ducts for the GDI engine exhaust exposure scenario (Figure 2). Increased peribronchial and perivascular inflammation was observed in HDM-allergic animals compared to non-allergic animals for all exposure scenarios (Figure 3). However, inflammation did not appear to be exposure dependent. No differences in parenchymal infiltrates were noted between exposure types.

Masson-Trichrome staining revealed no changes in collagen deposition between exposure types or from sensitisation with HDM (Figure S5). Clara cell numbers in the bronchial wall were elevated in HDM-allergic animals compared to non-allergic animals. No differences were noted between exposure types. Goblet cell concentrations within the airways of HDM-allergic animals were greater than those in non-allergic animals (Figure S6). No difference in goblet cell counts was observed between exposure groups.

### 3.6. Gene Expression

**Figure 4:**
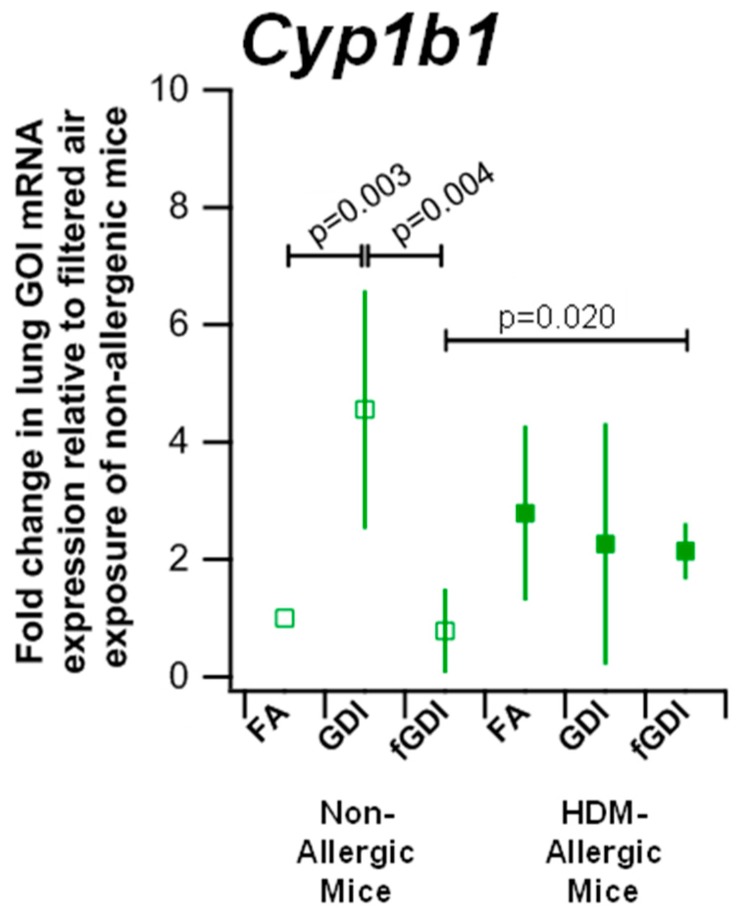
Enhanced expression of *Cyp1b1* following GDI engine exhaust exposure in non-allergic animals. *Cyp1b1* mRNA expression was measured in lung homogenates from naïve mice (non-allergic, open squares) and mice with airway hyperresponsiveness (HDM allergic, filled squares) exposed to HEPA filtered air (FA), GDI engine exhaust, or filtered GDI engine exhaust (fGDI). Genes of interest (GOI) transcript expression were normalized to *Ppia* expression and expressed as a fold change relative to the FA exposed non-allergic mice exposed. Means ± SDs are shown from 5 mice per group.

Cytochrome P450 1b1 mRNA expression was evaluated to examine PAH metabolism after exposure to GDI engine exhaust (Figure 4, Table S1). Elevated mRNA levels were observed in non-allergic animals exposed to GDI engine exhaust (4.56-fold change, SD = 2.03) compared to the HEPA filtered air control (*p* = 0.003), and f-GDI engine exhaust (0.78, SD = 0.72; *p* = 0.004). While, no difference was observed in *Cyp1b1* expression in HDM-allergic animals between the different exposure groups, HDM-allergic animals exposed to f-GDI engine exhaust exhibited elevated *Cyp1b1* expression (2.14, SD = 0.48) relative to non-allergic animals in the same exposure group (*p* = 0.020).

**Figure 5:**
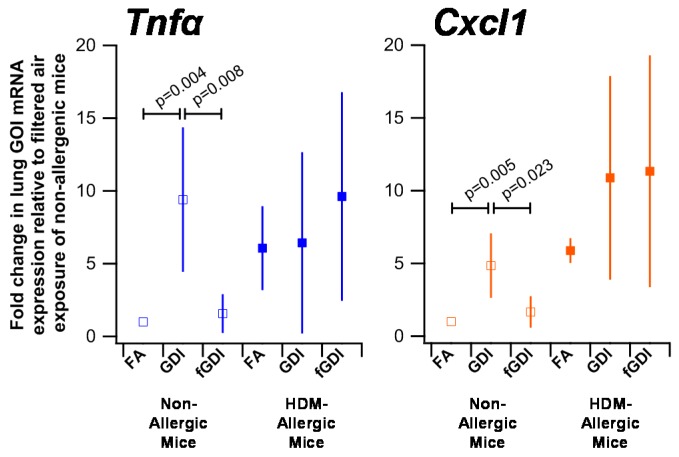
Airway inflammation induced following f-GDI engine exhaust exposure. *Cxcl1* and *Tnfα* mRNA expression was measured in lung homogenates from naïve mice (non-allergic; open squares) and mice with airway hyperresponsiveness (HDM allergic, filled squares) exposed to HEPA filtered air (FA), GDI engine exhaust, or filtered GDI engine exhaust (fGDI). Genes of interest (GOI) transcript expression were normalized to *Ppia* expression and expressed as a fold change relative to the non-allergic mice exposed to HEPA filtered air (saline-FA). Means ± SDs are shown from 5 mice per group.

*Tnfα* and *Cxcl1*, the murine homologue of the *Il8* transcript in humans, are markers of inflammation (Figure 5, Table S2). Baseline expression of both *Tnfα* and *Cxcl1* was increased in HDM-allergic animals compared to non-allergic animals. However, no difference was found across exposure groups for these two inflammatory markers in HDM-allergic animals. The gene expression of *Tnfα* was elevated in non-allergic mice exposed to GDI engine exhaust (9.40, SD = 4.97) compared to those exposed to HEPA filtered air (*p* = 0.004) and f-GDI engine exhaust (1.56, SD = 1.34; *p* = 0.008). Moreover, upregulated *Cxcl1* expression was found in non-allergic animals exposed to GDI engine exhaust (4.85, SD = 2.22; *p* = 0.005) relative to non-allergic animals exposed to HEPA filtered air and f-GDI engine exhaust (1.67, SD = 1.08; *p* = 0.023).

## 4. Discussion

The objective of this study was to examine the capacity for the gas and particle fractions of gasoline direct injection engine exhaust to induce respiratory responses in a mouse model. We evaluated the in vivo response to GDI engine exhaust in an allergic and non-allergic mouse model using pulmonary function tests, lung tissue histology, and genetic markers of PAH metabolism and inflammation in the lung. However, examination of the pulmonary immune response was out of the scope of this manuscript. To the best of our knowledge, this is the first study to publish results of the pulmonary effects of GDI engine exhaust emissions in English. Short-term exposure to particulate engine exhaust emissions induced upregulation of the inflammatory marker *Tnfα* in the airways of naïve mice, but not mice with hyperresponsiveness of the airways. In mice with airway hyperresponsiveness, results suggest a combination of the gas and particle fractions of the GDI engine exhaust may contribute to enhanced oxidative stress and airway resistance to methacholine.

Published murine in vivo studies have focused on diesel engine exhaust exposures. Some of these studies have used intratracheal instillation to examine the effect of diesel engine particulate matter on mice. The disadvantage to this exposure method is that the gaseous fraction of the exhaust is excluded in the resuspended particle solution. Other studies have used an inhalation exposure mode that more closely reflects realistic responses to diesel engine exhaust than instillation exposure scenarios [17]. This is the first murine inhalation study to evaluate the pulmonary effects of the gaseous and particulate fractions of GDI engine exhaust in an asthmatic model.

The intensity of perivascular and peribronchial cell infiltration was elevated in HDM-allergic animals compared to non-allergic animals, but no consistent increase was found for the engine exhaust exposure scenarios. The similar responses observed between exposure scenarios are likely a result of the short single exposure to diluted exhaust. Increasing the frequency or the duration of the exposure may contribute to a differentiated response across exposure groups.

We observed increased maximum total airway resistance in response to methacholine (100 mg/mL) for HDM-allergic animals exposed to GDI engine exhaust and f-GDI engine exhaust compared to their non-allergic counterparts. No difference was seen across non-allergic exposure groups. These results suggest that sensitisation with HDM enhances airway sensitivity to pollutant exposure. Previous studies evaluating asthmatic mouse models have looked at the airway resistance response to methacholine. Chew et al. [33] sensitised mice with ovalbumin using intraperitoneal injections and then challenged the animals with ovalbumin. These authors reported enhanced total airway resistance in the sensitized mice.

Total airway resistance following methacholine was increased for exposure to GDI and f-GDI engine exhaust compared to HEPA filtered air in HDM-allergic animals. We found no difference between the two engine exhaust exposure scenarios, which suggested a combined influence from the particle and gas fractions. Exposure of HDM-allergic animals to GDI engine exhaust further induced increased respiratory resistance in the central airways and peripheral tissue; however, similar effects were not apparent after exposure to only the gas fraction of the exhaust. These findings highlight the role of the particle fraction in this responsiveness of the peripheral and central airway tissues in HDM-allergic mice. It should be noted that we cannot definitively exclude the effect of the gas fraction. This is the first study to evaluate changes in lung mechanics attributable to the gas and particulate fractions of vehicle exhaust. Further studies would be warranted to evaluate the contribution of the gas fraction to this response.

Non-allergic animals exposed to GDI engine exhaust experienced increased levels of *Cyp1b1* compared to the HEPA filtered air and f-GDI engine exhaust scenarios. These results suggest that the particulate fraction of GDI engine exhaust may be responsible for the upregulation of *Cyp1b1* in these naïve animals. *Cyp1b1* has been reported to respond to PAH exposure, and the particle PAHs in the GDI engine emissions were dominated by high molecular weight species [34,35]. As the water solubility of PAHs decreases with increasing molecular weight, particle PAHs suggested to mediate the observed airway response were likely associated with the water insoluble particle fraction of GDI engine exhaust [36,37].

We also assessed *Tnfα* and *Cxcl1* mRNA expression as markers of airway inflammation. No difference in expression for both genes was observed in HDM-allergic animals across all exposure groups. This result was likely due to elevated basal levels of airway inflammation, which were supported by histology examination of the airways. *Tnfα* and *Cxcl1* upregulation were, however, found in non-allergic animals exposed to GDI engine exhaust compared to HEPA filtered air and f-GDI scenarios, suggesting that the inflammatory response in these naïve animals was likely induced by the particle fraction of the GDI engine exhaust. Parallel trends were found for *Cyp1b1* expression, which may indicate that particulate PAH emissions from the GDI engine could be attributable to this inflammatory airway response in non-allergic mice. An inflammatory airway response was also evaluated by Miyabara et al. [38] in non-allergic and ovalbumin-sensitized mice following inhalation exposure to diesel engine exhaust. In contrast to the present study, Miyabara et al. observed enhanced TNFα protein expression for their non-allergic mice exposed to diesel exhaust compared to filtered air but did not delineate if the response was attributable to the gas or particle fraction. Ovalbumin-allergic mice exposed to diesel engine exhaust were further reported to exhibit enhanced TNFα protein expression compared to the filtered air exposure. The contrasting results compared to the present study may be a result of the longer repeated exposure times tested by Miyabara et al. (12 h per day for five to six weeks), as well as compositional differences related to the PAH phase partitioning between the diesel and GDI engine exhaust exposure scenarios [23].

The mean formaldehyde concentration measured in our GDI raw engine exhaust was 0.87 ppm (SD = 0.005), which approaches the short-term occupational exposure limit of 1 ppm set by the Ontario Ministry of Labour [39]. Benzene and toluene were present in the engine exhaust at concentrations of 0.06 ppm (SD = 0.02) and 0.6 ppm (SD = 0.05). These levels are below the Ontario Ministry of Labour’s Time Weighted Average maximums of 0.5 ppm for benzene and 20 ppm for toluene [39]. However, mice have increased susceptibility to airborne toxins due the smaller size of their lungs and resultant increase in surface area to volume ratio, as well as the higher breathing rate observed compared to humans.

A limitation of this study is that multiple consecutive exposure scenarios and multiple sampling times post-exposure were not measured due to limitations in place by the animal facility. The time that mice could remain alive for post-exposure was limited. These are promising avenues for future studies.

## 5. Conclusions

As GDI engines become predominant in the global vehicle fleet, understanding the impact of GDI engine exhaust on respiratory health becomes increasingly important for the development of new emission standards. GDI engines achieve improved fuel economy compared to the traditional port injection engine design; however, these engines release increased PM emissions. In this study, the particle fraction of GDI raw uncatalysed engine exhaust was found to induce increased expression of genes associated with airway inflammation (*Tnfα*, *Cxcl1*) in non-allergic mice following a short-term exposure period. Parallel upregulation of *Cyp1b1*, a gene related to PAH metabolism, suggested the high molecular weight PAHs associated with the particulate matter engine emissions may be attributable to this inflammatory airway response. In HDM-allergic mice, enhanced resistance in the central airways and dampening of the peripheral tissues in response to methacholine was attributable to exposure to the particle fraction of GDI raw engine exhaust. A combination of pollutants in the gases and particle fractions was found to drive increased resistance across the total airway. The adverse pulmonary responses induced by raw GDI engine exhaust in non- and HDM-allergic mice highlight the need to decrease emissions with priority placed on PAH compounds, as current catalytic converters may not effectively remove these compounds.

## Figures and Tables

**Table 1:** Exposure to GDI engine exhaust increases total respiratory system resistance. The maximum resistance achieved in response to 100 mg/mL methacholine across the total airway, as well as the peripheral and central tissues for non- and HDM-allergic animals, is summarised as means ± SDs for 8 to 9 mice per group.

Title	Exposure Group	Non-Allergic Mice	HDM-Allergic Mice
Total Airway Resistance Max	FA	6.34 ± 2.34	10.15 ± 3.50
	GDI	7.19 ± 4.26	14.78 ± 2.32
	fGDI	7.63 ± 2.48	14.49 ± 2.52
Central Tissue Max	FA	2.01 ± 0.41	2.51 ± 1.08
	GDI	1.83 ± 0.64	3.99 ± 1.11
	fGDI	2.28 ± 0.73	2.55 ± 0.76
Peripheral Tissue Max	FA	31.05 ± 15.93	48.84 ± 16.05
	GDI	29.81 ± 15.22	95.56 ± 30.90
	fGDI	36.45 ± 12.41	78.94 ± 33.64

## Supplementary Materials

The following are available online at http://www.mdpi.com/1660-4601/15/3/429/s1, Figure S1: allergic sensitisation is exacerbated in HDM-allergic animals exposed to GDI exhaust, Figure S2: total airway resistance response to increasing methacholine doses, Figure S3: central airway tissue resistance response to increasing methacholine doses, Figure S4: resistance response in the peripheral airway tissues to increasing methacholine doses, Figure S5: airway fibrosis was unchanged by GDI engine exhaust exposures, Figure S6: enhanced mucus hyperplasia in HDM-sensitised animals, Table S1: Summary of maximum resistance achieved in response to 100 mg/mL methacholine across the total airway as well as the peripheral and central tissues for non- and HDM-allergic animals. Means ± SEM are shown from 8 to 9 mice per group, Table S2: Summary of *Cyp1b1*, *Cxcl1* and *Tnfa* mRNA expression measured in lung homogenates from naïve mice (non-allergic) and mice with airway hyperresponsiveness (HDM allergic) exposed to HEPA filtered air (FA), GDI engine exhaust or filtered GDI engine exhaust (fGDI). Genes of interest (GOI) transcript expression were normalized to Ppia expression and expressed as a fold change relative to the non-allergic mice exposed to HEPA filtered air (saline-FA). Means ± SEM are presented from 5 mice per group.
