# Supplementary material for: Comparison of Airway Responses Induced in a Mouse Model by the Gas and Particulate Fractions of Gasoline Direct Injection Engine Exhaust
Source: Int J Environ Res Public Health. 2018 Mar 1;15(3):429. doi: 10.3390/ijerph15030429 (PMC5876974; doi:10.3390/ijerph15030429)
Supplement: Supplementary file 1 [file ijerph-15-00429-s001.pdf]

SUPPLEMENTARY MATERIAL

Comparison of airway responses induced in a mouse model by the gas and particulate fractions of gasoline direct injection engine exhaust

Caitlin L. Maikawa
Environmental Health Sciences, University of Massachusetts, Amherst, MA, USA.
Present address: Materials Science & Engineering, Stanford University, Stanford, CA, USA.
Email: cmaikawa@stanford.edu

Naomi Zimmerman
Mechanical Engineering, University of British Columbia, Vancouver, British Columbia, Canada.
Email: nzimmerman@mech.ubc.ca

Manuel Ramos
Mechanical and Industrial Engineering, University of Toronto, Toronto, Ontario, Canada.
Email: manuel.ramos@mail.utoronto.ca

Mittal Shah
Institute of Orthopaedics and Musculoskeletal Sciences, University College London, Royal National Orthopaedic Hospital, London, United Kingdom
Email: mittal.shah@ucl.ac.uk

James S. Wallace
Mechanical and Industrial Engineering, University of Toronto, Toronto, Ontario, Canada.
Email: wallace@mie.utoronto.ca

Krystal J. Godri Pollitt
Corresponding Author
Environmental Health Sciences, University of Massachusetts, 686 North Pleasant Street, Goessmann Laboratory Room 175, Amherst, MA, 01003, USA.
Tel.: +1 413 545 1778.
Email: kpollitt@umass.edu

Table S1. Summary of maximum resistance achieved in response to 100 mg/mL methacholine across the total airway as well as the peripheral and central tissues for non- and HDM-allergic animals. Means ± SEM are shown from 8 to 9 mice per group.

	Exposure Group	Non-Allergic Mice	HDM-Allergic Mice
Total Airway Resistance Max	FA	6.34 ± 0.29	10.15 ± 0.44
	GDI	7.29 ± 0.49	13.93 ± 0.44
	fGDI	13.88 ± 0.33	7.63 ± 0.28
Central Tissue Max	FA	2.01 ± 0.05	2.51 ± 0.14
	GDI	1.83 ± 0.09	3.68 ± 0.18
	fGDI	2.28 ± 0.08	2.65 ± 0.09
Peripheral Tissue Max	FA	31.05 ± 1.99	53.64 ± 2.77
	GDI	29.81 ± 2.17	89.30 ± 4.67
	fGDI	36.45 ± 1.28	78.94 ± 3.74

Table S2. Summary of *Cyp1b1*, *Cxcl1* and *Tnfa* mRNA expression measured in lung homogenates from naïve mice (non-allergic) and mice with airway hyperresponsiveness (HDM allergic) exposed to HEPA filtered air (FA), GDI engine exhaust or filtered GDI engine exhaust (fGDI). Genes of interest (GOI) transcript expression were normalized to Ppia expression and expressed as a fold change relative to the non-allergic mice exposed to HEPA filtered air (saline-FA). Means ± SEM are presented from 5 mice per group.

	Exposure Group	Non-Allergic Mice	HDM-Allergic Mice
Cyp1b1	FA	1	2.79 ± 0.37
	GDI	4.56 ± 0.51	2.27 ± 0.51
	fGDI	0.78 ± 0.17	2.14 ± 0.12
Cxcl1	FA	1	5.88 ± 0.21
	GDI	4.85 ± 0.55	10.89 ± 1.40
	fGDI	1.67 ± 0.27	11.34 ± 1.99
Tnfa	FA	1	6.06 ± 0.72
	GDI	9.40 ± 1.24	6.43 ± 1.56
	fGDI	1.56 ± 0.33	9.62 ± 1.79

HDM-specific and total IgE and IgG

We examined the airway response in HDM-allergic animals to GDI engine exhaust. Total and HDM-specific IgE and IgG proteins were measured to evaluate the effectiveness of the HDM-sensitisation at eliciting an allergic response, and whether that response was associated with the HDM allergen. An anitgen-capture enzyme-linked immunosorbent assay (ELISA) was used to evaluate IgE levels in mouse serum samples. Maxi-Sorp plates (NUNC Brand Products, Denmark) were prepared with a HDM coating. Manufacturer directions for the biotin-anti mouse total IgE ELISA kit (Biolegend, CA, USA) were then followed. IgE and IgG were measured by absorbance at 450nm and concentrations were assessed using a six-point calibration curve (0.156-5 ng/mL). Analysis was conducted on five samples from each of the non-allergic and HDM-allergic animal exposure groups.

Elevated total IgE, total IgG, HDM-specific IgE, and HDM specific IgG levels were observed in HDM-allergic animals exposed to HEPA filtered air, GDI engine exhaust and filtered GDI engine exhaust compared to the non-allergic mice exposed to HEPA filtered air ($p<0.05$) (Figure S1). HDM-allergic animals experienced higher levels of total IgE, total IgG, HDM-specific IgE, and HDM specific IgG than non-allergic mice in the GDI engine exhaust and filtered GDI engine exhaust exposure groups. Enhanced IgE and IgG levels in HDM- allergic mice confirm that sensitisation of mice produced an allergic response.

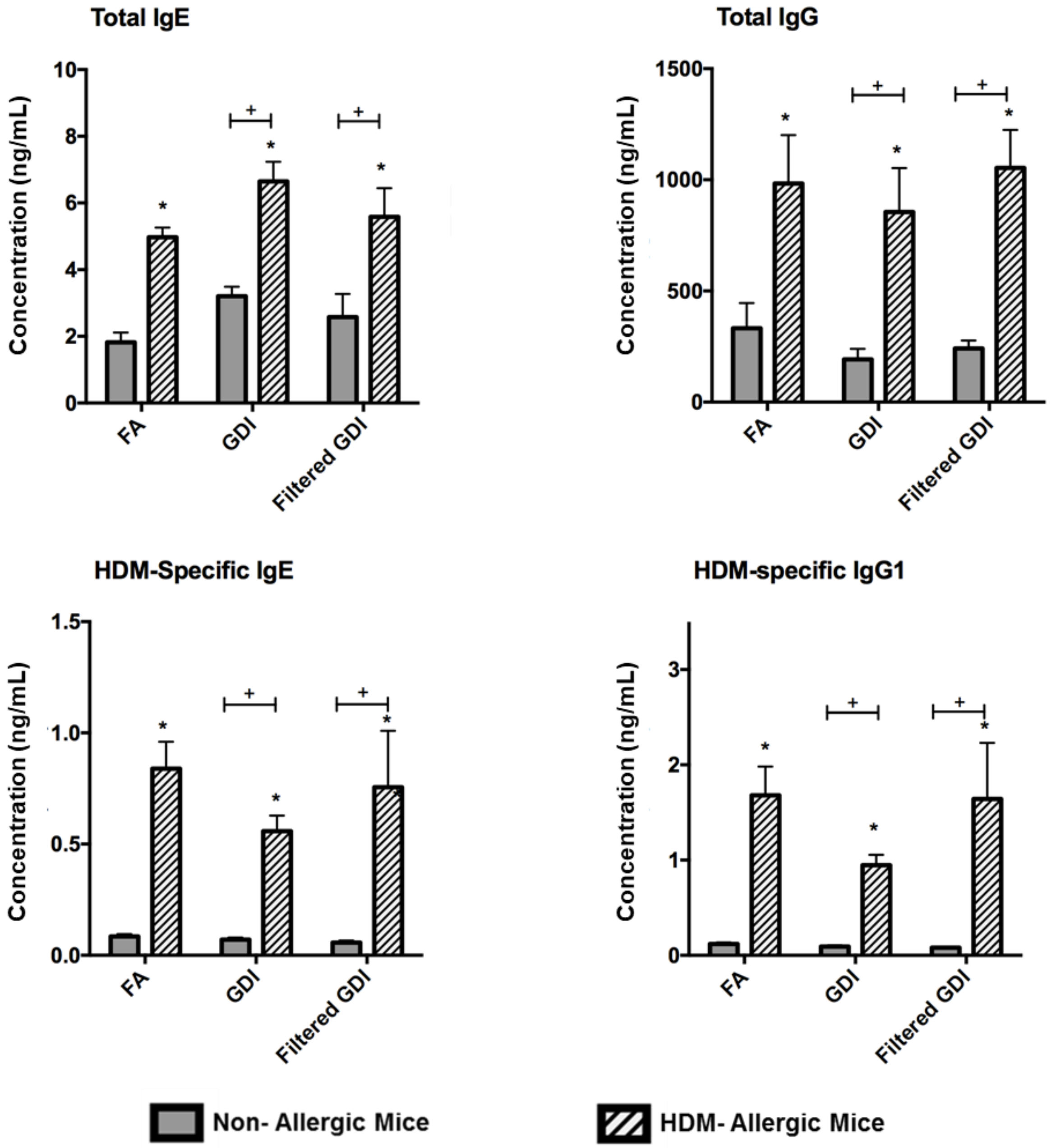

Figure S1: Allergic sensitisation is exacerbated in HDM-allergic animals exposed to GDI exhaust. Filtered GDI engine exhaust exposure in non-allergic animals did not increase total IgE levels compared HEPA filtered air (FA) exposures. Repeated exposure to HDM was confirmed to induce allergic sensitisation. Significantly increased HDM-specific IgE levels in HDM-treated animals following GDI engine exhaust (GDI) exposures suggest exhaust pollutants are capable of exacerbating allergic sensitisation. * $p<0.05$ relative to FA exposed non-allergic mice; + $p< 0.05$ difference between indicated groups.

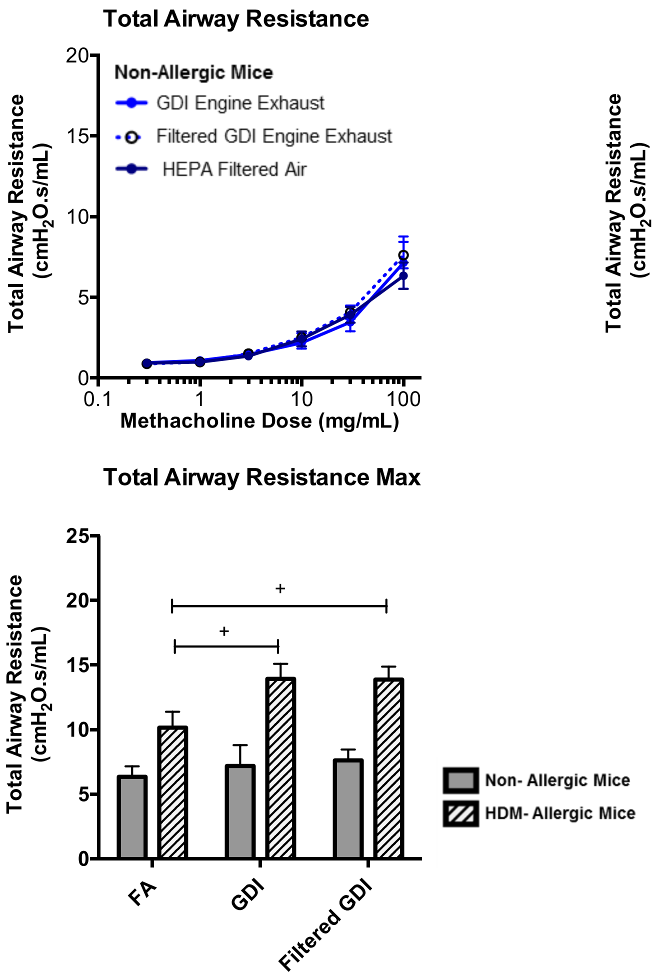

Figure S2: Total airway resistance response to increasing methacholine doses. Increased methacholine dose had no effect on airway resistance between exposure types in non-allergic mice (upper left). Airway resistance was increased for HDM-allergic animals exposed to GDI engine exhaust compared to HEPA filtered air and filtered GDI engine exhaust exposure in HDM-allergic animals (upper right). Maximal total resistance for HDM-allergic animals exposed to GDI and filtered GDI engine exhaust exposures was increased compared to the HEPA filtered air exposure (FA) (lower left). * $p<0.05$ relative to FA exposed non-allergic mice; + $p< 0.05$ difference between indicated groups; +++ $p<0.001$ difference between indicated groups.

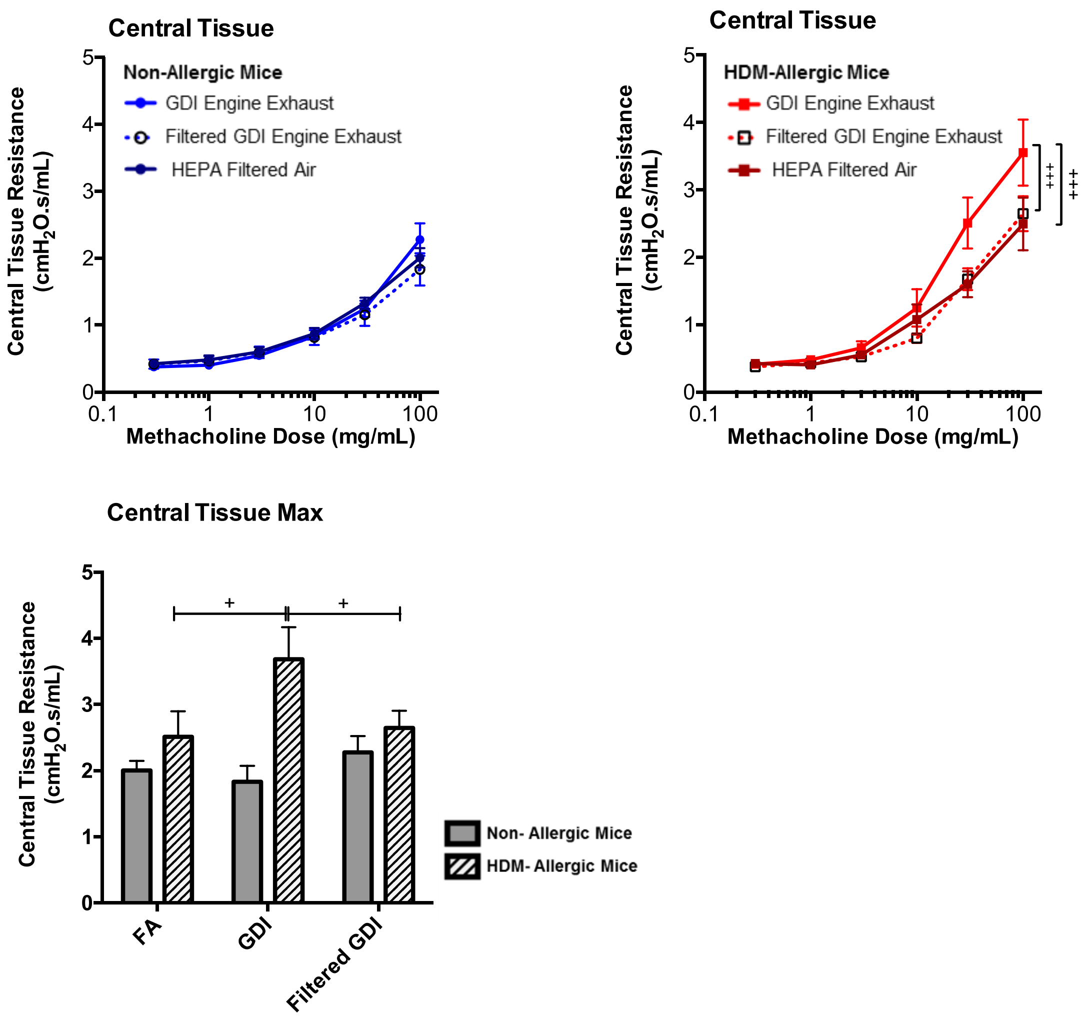

Figure S3: Central airway tissue resistance response to increasing methacholine doses. Enhanced central airway resistance was observed with increasing doses methacholine for GDI engine exhaust exposure in HDM-allergic animals. Maximal central airway resistance for HDM-allergic animals exposed to GDI engine exhaust was significantly increased compared to the HEPA filtered air (FA) and filtered GDI engine exhaust exposures. * $p<0.05$ relative to FA exposed non-allergic mice; + $p< 0.05$ difference between indicated groups; +++ $p<0.001$ difference between indicated groups.

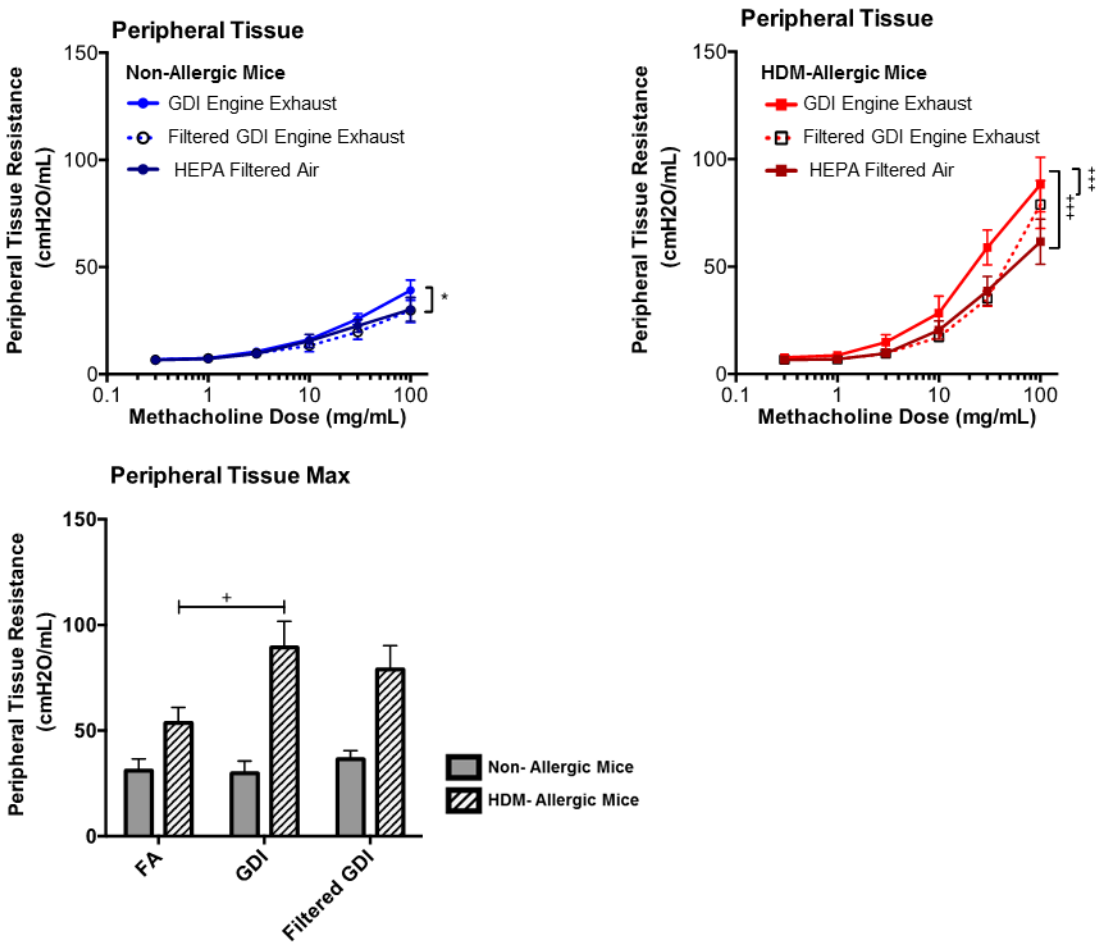

Figure S4: Resistance response in the peripheral airway tissues to increasing methacholine doses. Enhanced peripheral tissue resistance was observed with increasing doses methacholine for GDI and filtered GDI engine exhaust exposures in HDM-allergic mice. Maximal total resistance for GDI and filtered GDI engine exhaust exposure was significantly increased compared to the HEPA filtered air exposure (FA). * $p<0.05$ relative to FA exposed non-allergic mice; + $p< 0.05$ difference between indicated groups; +++ $p<0.001$ difference between indicated groups.

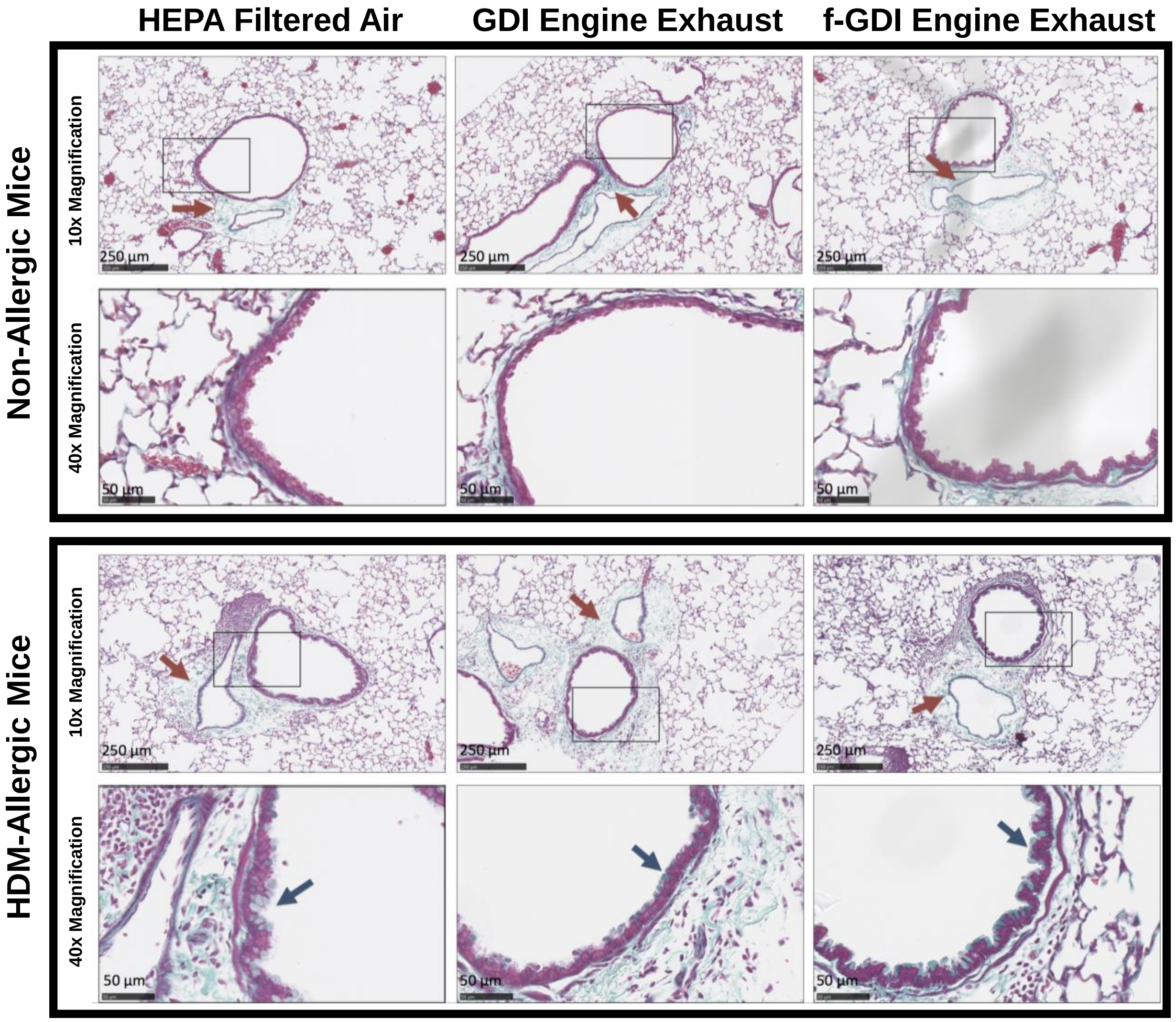

Figure S5: Airway fibrosis was unchanged by GDI engine exhaust exposures. Mason Trichrome staining indicated unchanging sub-epithelial and vascular collagen deposition (orange arrows, Rows 1 and 3) between exposure types and sensitisation groups. Increased Clara cell counts were observed in the airways of HDM-allergic animals (blue arrows, Rows 2 and 4). High power images (40x magnification, Rows 2 and 4) are representative of the boxed regions in the low power images (10x magnification, Rows 1 and 3).

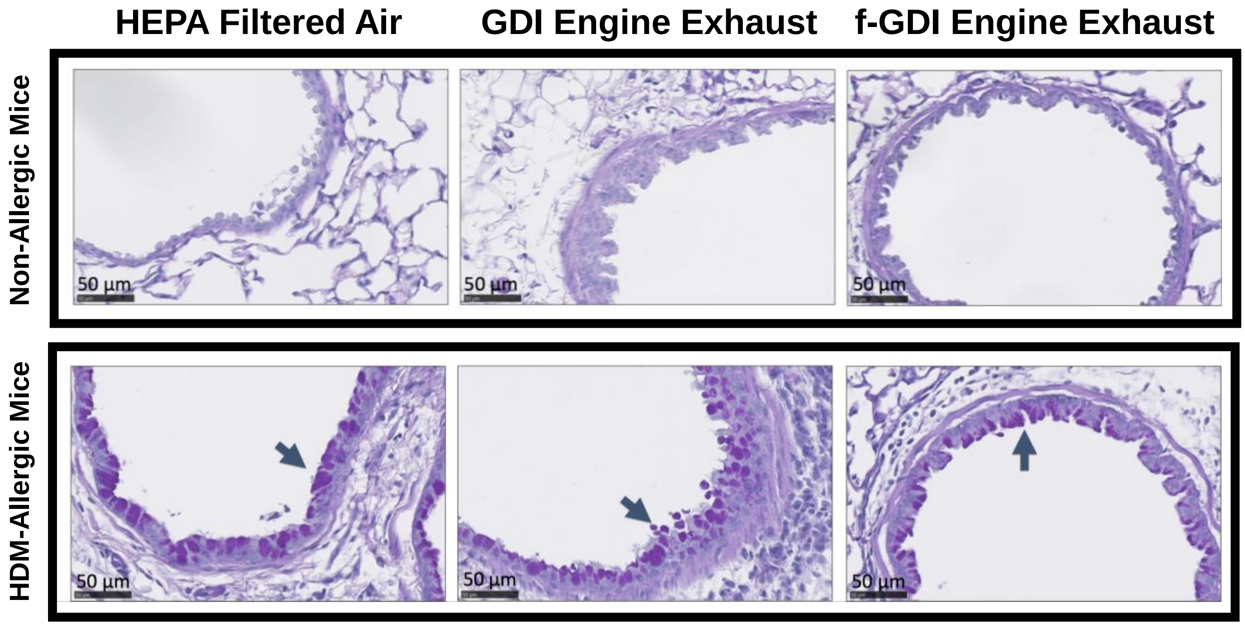

Figure S6: Enhanced mucus hyperplasia in HDM-sensitised animals. Periodic acid-Schiff-diastase staining indicated increased goblet cell counts in HDM-allergic mice as compared non-allergic mice. Regions of goblet cells are indicated by blue arrows. Images were shown at 20x magnification.

References

Brandt EB, Kovacic MB, Lee GB, Gibson AM, Acciani TH, Le Cras TD, et al. Diesel exhaust particle induction of IL-17A contributes to severe asthma. Journal of Allergy and Clinical Immunology 2013; 132: 1194-1204.e2.

Maes T, Provoost S, Lanckacker EA, Cataldo DD, Vanoirbeek JAJ, Nemery B, et al. Mouse models to unravel the role of inhaled pollutants on allergic sensitization and airway inflammation. Respiratory Research 2010; 11.

McCreanor J, Cullinan P, Nieuwenhuijsen MJ, Stewart-Evans J, Malliarou E, Jarup L, et al. Respiratory effects of exposure to diesel traffic in persons with asthma. New England Journal of Medicine 2007; 357: 2348-2358.

Miyabara Y, Ichinose T, Takano H, Lim HB, Sagai M. Effects of diesel exhaust on allergic airway inflammation in mice. Journal of Allergy and Clinical Immunology 1998; 102: 805-812.

Riedl M, Diaz-Sanchez D. Biology of diesel exhaust effects on respiratory function. Journal of Allergy and Clinical Immunology 2005; 115: 221-228.

Steenhof M, Mudway IS, Gosens I, Hoek G, Godri KJ, Kelly FJ, et al. Acute nasal pro-inflammatory response to air pollution depends on characteristics other than particle mass concentration or oxidative potential: The RAPTES project. Occupational and Environmental Medicine 2013; 70: 341-348.
